# Tracing Nitrogen Flows Associated with Beef Supply Chains: A Consumption-Based Assessment

**DOI:** 10.1021/acs.est.4c01651

**Published:** 2024-08-02

**Authors:** Anaís Ostroski, Oleg A. Prokopyev, Vikas Khanna

**Affiliations:** † Department of Civil and Environmental Engineering, University of Pittsburgh, 742 Benedum Hall, 3700 O’Hara Street, Pittsburgh, Pennsylvania 15261, United States; ‡ Department of Industrial Engineering, University of Pittsburgh, 1025 Benedum Hall, 3700 O’Hara Street, Pittsburgh, Pennsylvania 15261, United States; § Department of Chemical and Petroleum Engineering, University of Pittsburgh, 3700 O’Hara Street, Pittsburgh, Pennsylvania 15261, United States

**Keywords:** beef production, nitrogen footprint, optimization, supply chain, network analysis

## Abstract

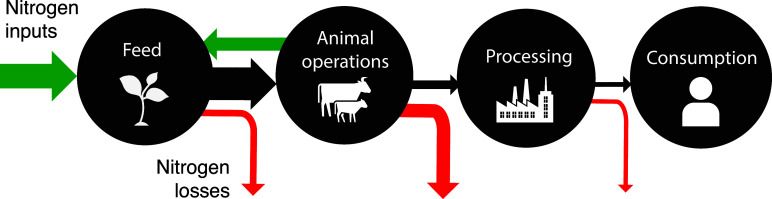

While highly connected food chains provide numerous benefits, they lack traceability and transparency. As such, understanding the spatial heterogeneity in their environmental burdens is critical for targeted interventions. This is especially important for nutrient-related impacts such as nitrogen since the release of reactive nitrogen has been linked to loss of biodiversity and decrease in water quality in different parts of the world. Animal feed production is heavily dependent on synthetic fertilizers, and the consumption of beef products, in particular, is associated with high nitrogen footprints. Although there is a rich body of work on nutrient footprints of beef production, there is a gap in understanding the spatial distribution of the nutrient releases throughout the beef supply chain in the U.S. We present an optimization-based framework to trace supply chain networks of beef products at the county level. Using publicly available data, we construct a weighted network of nutrient flows based on mass balance, including synthetic fertilizers, manure production, and crop uptake and residues. The results show that beef consumption in a county can be associated with nitrogen losses in hundreds of counties. One year worth of beef consumption in the United States released approximately 1.33 teragrams (Tg) of N to the environment, and most of it as diffuse pollution during the feed production phase. Analysis also revealed the huge disparity between consumption-based and production-based impacts of beef and the need for considering consumption-based accounting in discourse around the environmental sustainability of food systems.

## Abstract 2

We present a framework to trace beef supply chains from feed production to consumption at the county level and report associated nitrogen flows.

## Introduction

Although nitrogen is abundant and makes up the majority of earth’s atmosphere, it is mostly in the form of dinitrogen (N_2_) which has a strong triple-bond that is very difficult to break apart.^1^ On the other hand, most organisms need a reactive form of nitrogen (Nr) to sustain life. Some nitrogen-fixing microorganisms can convert molecular nitrogen into more useful forms, and they have been controlling most of the nitrogen cycle for hundreds of millions of years.^1^ The Haber–Bosch process for ammonia production fundamentally revolutionized the synthetic fertilizer industry and helped sustain food production for a growing population.^2^ Nonetheless, human and industrial activities have since altered the nitrogen cycle, and reactive nitrogen now accumulates in the environment.^3−7^ Reactive nitrogen can keep causing multiple effects on the environment until it is converted back to nonreactive form through a process called nitrogen cascade.^8^ These reactive nitrogen forms have been linked to impacts on human health, via air pollution and excess intake of nitrogen from foods and water; on aquatic systems, via acidification and eutrophication; on terrestrial systems, via reduction of plant species richness; and on climate change, through nitrous oxide (N_2_O) formation—a greenhouse gas, as well as ground-level ozone formation.^9^

With agriculture becoming a major driver of alterations in nitrogen cycle, food consumption patterns play an increasingly important role in nitrogen flows in the environment.^10,11^ More specifically, protein-rich foods have been linked to increases in anthropogenic nitrogen inputs.^12^ A large share of arable land is dedicated to the production of feed, much of which relies on synthetic fertilization, especially crops such as corn used to produce concentrate feed.^13^ Approximately 40% of corn produced in the United States is used as feed,^14^ and this is especially relevant because about half of nitrogen emissions in beef supply chains occur during feed production.^15^ While nitrogen is an essential constituent of proteins because they make up amino acids,^16^ the conversion from nitrogen intake to animal protein is inefficient, with cattle retaining only about a quarter of the nitrogen it consumes.^10,17^ Large ruminants have lower nitrogen use efficiencies (NUE) than other livestock, such as broiler poultry systems with NUE ranging from 30 to 60% or industrial pigs with NUE between 30 and 40% in comparison to 25–30% of feedlot beef cattle. This is highly influenced by the feed conversion rates because cattle need as much as 25 kg of feed per kg of edible weight produced, while poultry requires about 4.5 kg and pigs 9.4 kg.^18^ Protein conversion efficiency in beef cattle was estimated to be as low as 4%, in comparison to 10% for pigs and 20% for poultry.^18^ Nitrogen is released to the environment across the life cycle of animal-based products through multiple pathways and locations. Feed production, calf production, cattle feeding, and slaughtering often occur in distant, industrially specialized areas. Further, the geographical concentration of beef production results in the production of manure in quantities that surpass the absorptive capacities of surrounding land, effectively creating a unidirectional flow of nutrients and hindering the ability to set circular systems for recycling manure and nutrients.^15,19^

The overall impact of beef production on nitrogen cycles has been well illustrated by nitrogen footprinting studies.^20−26^ Assessments characterizing nitrogen use of specific products at the national level help monitor country-level performance of sustainability goals.^27^ However, assessing nitrogen losses in specific compartments (water, soil, atmosphere) and forms of reactive nitrogen that are released and by which mechanisms at the supply chain level is essential for designing better management practices. Typically, nitrogen budgeting is employed to account for inputs, outputs, retention, and transformations using the mass-balance approach in combination with emission factors.^28^ Uwizeye et al.^29^ provided a comprehensive assessment of nitrogen emissions of globalized livestock supply chains with a disaggregated and spatially explicit approach to quantify nitrogen flows of production systems and livestock categories. McLellan et al.^30^ argued that nitrogen balances for farms and supply chains provide the appropriate level of information for targeted practical implementations and for defining local safe operating regions.^24^ These studies significantly improved our understanding of nitrogen impacts of food products. However, there still exists a lack of subnational scale data to spatially link production stages, including feed and animal movements, and systematically attribute the nitrogen footprint associated with the consumption of animal products to their production impacts in distant location. Consumption-based accounting can provide a complementary perspective to production-based footprinting as well as highlight how consumption patterns affect nitrogen flows in distant locations. This is a critical knowledge gap because food supply chains have become increasingly complex and are characterized by long distances between stages, increasing the disparity between production- and consumption-based impacts.

We fill the aforementioned knowledge gap by modeling county-level beef supply chain flows using an optimization-based approach and couple it with nitrogen budgeting to account for nitrogen losses from production to consumption. The understanding of these movements and associated nitrogen balances is essential for the characterization of nitrogen sources and sinks to inform targeted interventions and identify hotspots. In addition, the model allows for a comprehensive representation of resources and emissions previously unattainable with aggregated trade data and input-output tables. It is the first detailed accounting of nitrogen flows at the county level for beef supply chains from feed production to consumption in the United States. The framework leverages agricultural production data sets, surveys on feed requirements and cattle production practices, as well as nitrogen inputs and emission factors from the literature to map the spatial distribution of nitrogen impacts associated with beef consumption. The results provide an unprecedented level of detail in mapping nitrogen emissions and use efficiency across the beef supply chain, providing a new perspective on how the environmental impacts are spatially distributed in relation to consumption patterns.

## Methodology

### Network Construction

We leverage a previously developed county-level beef network model for tracing flows throughout 4 major steps in beef supply chain: (i) ranch, (ii) feedlot, (iii) slaughter, and (iv) consumption.^31^ We refer to ranch as the location of animals during the calf production, or cow-calf phase, and feedlot as the location of animals during both backgrounding and finishing phases. In addition, mother cows in the cow-calf phase are referred to as beef cows, and cattle ready at the end of the finishing phase are referred to as fed cattle. This model was devised to trace animal and meat movements in the United States. Further details of the optimization model are described in Ostroski et al.^31^ This previous model assumed that animal feed consumed was sourced from the same county as animal operations, with similar feed intake rations published by Statistics Canada.^32^ This is a major shortcoming in attempts to quantify nitrogen releases from animal feed production. As such, an extended beef network that considers spatially explicit accounting of animal feed sources was deemed necessary and is described next.

Region-specific feed intake of beef cattle has been characterized through a series of surveys by Asem-Hiablie et al.^33−36^ These surveys also provide parameters of production practices, such as the number of days an animal spends in ranches or feedlots, initial and final weight in each phase, and percentage of animals that are directed to the intermediary phase (stocker/backgrounding). The feed ratio for feedlot cattle includes corn silage, corn grain, distiller’s grain, alfalfa hay, and “other” (unspecified) as a percentage of dry matter (DM) intake. For ranches, the amount of feed was estimated based on stocking rate (defined as the amount of land per animal), purchased forage, and purchased concentrate given in kilograms of dry matter per animal per day and dry matter intake values from Rotz et al.^37^ Data on breakdown of amount of feed for calves and for beef cows in the cow-calf operation are not readily available. As such, we allocate the feed based on DM intake calculated based on net energy for growth. More information on the feed requirements can be found in the Supporting Information (SI). By combining this information with the agricultural census and the feed-food-fuel allocation by EarthStat,^38,39^ we link feed products to cattle ranches and feedlots via an optimization problem to minimize purchase and transportation costs as explained next.

Transportation costs for feed were derived based on estimates by Gonzales et al.^40^ They describe that the truck transportation cost per unit weight of feed transported was proportional to the distance (*d*) between counties. The distances were obtained from the National Bureau of Economic Research as great-circle distances using the Haversine formula.^41^ We adjusted these distances by multiplying by a factor of 1.21 after comparison with samples from Google Maps to obtain more accurate values for ground transportation. We only chose truck transportation because the majority of grains (around 80%) and distiller’s grains are transported by truck in the domestic market.^42,43^

Mathematically, we obtained the following optimization model
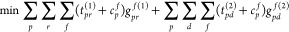
1a
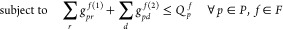
1b
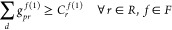
1c
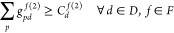
1d
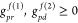
1e

The sets, parameters, and decision variables are as follows

**Sets**

*P* set of feed-producing counties

*F* set of feed types

*R* set of ranch (cow-calf) counties

*D* set of feedlot counties

**Parameters**

*t*_*pr*_^(1)^ cost to transport feed between counties *p* and *r*

*t*_*pd*_^(2)^ cost to transport feed between counties *p* and *d*

*c*_*p*_^*f*^ cost to purchase feed *f* from county *p*

*Q*_*p*_^*f*^ production capacity of feed *f* in county *p*

*C*_*r*_^*f*(1)^ demand of feed *f* in ranch county *r*

*C*_*d*_^*f*(2)^ demand of feed *f* in feedlot county *d*

**Decision variables**

*g*_*pr*_^*f*(1)^ flow of feed *f* from county *p* to ranch county *r*

*g*_*pd*_^*f*(2)^ flow of feed *f* from county *p* to feedlot county *d*

Equation 1a is the objective function to minimize the total transportation and purchase costs of feed to the ranch and feedlot. Constraint 1b imposes the appropriate upper bound on the feed production capacity at county *p* according to the 2017 agricultural census. Constraints 1c and 1d ensure that the amount of feed meets requirements in ranches and feedlots, respectively. Constraint 1e ensures that flows are non-negative. The obtained optimization model is a linear program (LP)^44^ and was solved with Gurobi version 9.1.0 via its Python package with dual simplex method.

### Quantifying Nitrogen Flows

**Figure 1:**
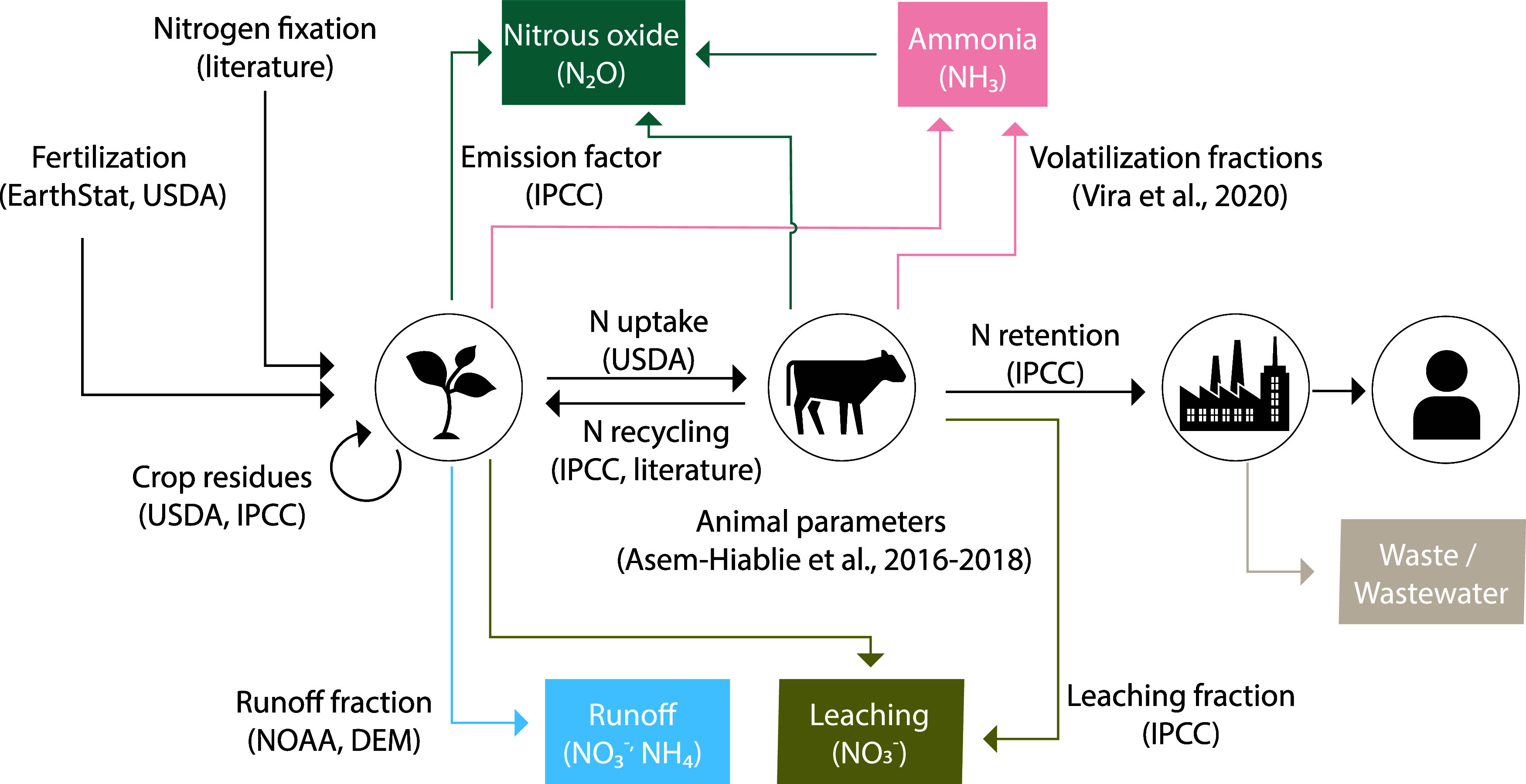
Schematic representation of the nitrogen flow methodology and the data sources used in this study.

Nitrogen flows in this work are estimated based on the methodologies developed by IPCC^45^ and Uwizeye.^15^ The overall schematic of nitrogen accounting is shown in Figure 1. The methodology is based on material flow considering inputs (fertilization, crop residues, biological fixation) and outputs (plant uptake, nitrogen loss, and nitrogen balance in the soil). The methods were applied for each module, representing major stages in the beef supply chain: (i) feed, (ii) animal, (iii) processing, and (iv) consumption. Each module also takes the beef network as input, and thus, the nitrogen retained from the previous module. A network whose edge weights represent the nitrogen retained and nitrogen emissions for each node in every stage are results of this framework.

In the feed module, we utilize crop-specific fertilization data from Earthstat^38,39^ and USDA.^46^ The EarthStat data is available at 10 km resolution and was upscaled to county level and adjusted according to 2017 state-level totals. Average plant nitrogen uptake was obtained from the USDA Crop Nutrient Tool^47^ and crop residues were calculated using IPCC^45^ parameters. Biological fixation was considered an input in soybeans^48^ and alfalfa.^49^

In the animal module, nitrogen inputs are estimated according to the phases of cattle production and specific feed intake. The nitrogen intake is the sum of nitrogen incoming in each animal phase from the feeds in the feed network, plus the estimated nitrogen content of additional, unspecified feeds (e.g., mineral supplements and others) assumed to be 2.16%.^50^ Nitrogen excretion is estimated with a Tier 2 method^45^ as the difference between intake and retention. Nitrogen retention is calculated based on the daily net energy for growth (NE_g_) for calves, backgrounding, and finishing. Beef cows were assumed to maintain body weight, and the default value of 7% nitrogen retention was assumed.^45^
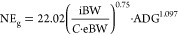
2where iBW is the initial body weight (kg), eBW is the final body weight (kg), ADG is the average daily gain (kg/day), and *C* is a coefficient of 0.9 which is the average between the values for heifers (females), of 0.8 and steers (neutered males), of 1.0. The daily nitrogen retention was calculated as
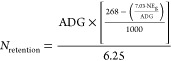
3Nitrogen losses are calculated based on the fractions that are lost via direct nitrous oxide, volatilization, runoff, leaching, and indirect nitrous oxide, and the respective emission factors. In the feed module, direct nitrous oxide emissions are calculated as
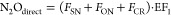
4where *F*_SN_ is the amount of fertilizer applied to soil (kg N/year), *F*_ON_ is the amount of manure applied to soil (kg N/year), *F*_CR_ is the annual amount of N in crop residues (above-ground and below-ground) returned to soils (kg N/year), and EF_1_ is the emission factor for nitrous oxide. Since data for manure application for specific crops at the county level are unavailable, it is assumed to be zero with the exception of manure deposited on pastureland. However, we carried out a sensitivity analysis to understand how manure application rates impact nitrogen emissions. In the animal module, nitrogen emissions are calculated first on a per animal per day basis for each phase. The nitrous oxide is estimated as
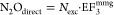
5where *N*_exc_ is the daily nitrogen excretion (kg N/day) and EF_3_^mmg^ is the emission factor for manure management mmg, assumed to be 0.004 for pasture/range paddock and 0.02 for drylot.^51,52^

Spatially explicit volatilization fractions for fertilizer, grazing, and manure management were obtained from Vira et al.^53^ In the feed module, fertilizer is the only nitrogen input assumed to emit ammonia through volatilization. Therefore, it was calculated as the product between the application rate and volatilization fraction. Similarly, in the animal module, ammonia emissions from volatilization were the product of excretion and volatilization fractions specific for grazing in cow-calf operations and manure management in feedlot operations. The manure management information was obtained from the national inventory of ammonia emissions from animal operations by the EPA.^54^ It was assumed that cow-calf operations manure management is pasture/range paddock and, for feedlots, a combination of drylot and solid storage.

In the IPCC guidelines,^45^ runoff and leaching are aggregated with one combined fraction and emission factor. We adopted the approach outlined in Uwizeye et al.^29^ to quantify nitrogen losses via runoff and leaching separately. Runoff is estimated using runoff fractions as a function of the maximum surface runoff for slope classes^15,55^ from the digital elevation model by Shuttle Radar Topography mission at 30 m scale, and precipitation surplus acquired from NOAA and USGS. Leaching was calculated as a function of runoff fraction, considering a maximum loss of runoff and leaching of 24%.^45^
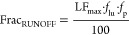
6
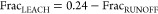
7where LF_max_ is the maximum surface runoff for different slope classes, *f*_lu_ is the reduction factor for land use or crop, and *f*_p_ is the reduction factor for precipitation.^15,55^ In the feed module, all nitrogen inputs were assumed to be susceptible to runoff and leaching. Runoff was assumed to be negligible in feedlot operations due to runoff estimates being small (2%,^50^ or between 2 and 4% with runoff and leaching combined^56^) and the assumption that runoff is collected in manure management, such as storage ponds, with very limited data on nitrogen loss from the treatment process.^54^ Indirect nitrous oxide emissions were also assumed to occur with emission factor EF_4_ of 0.01 for indirect emissions from volatilized portion, and EF_5_ of 0.011 from runoff and leaching portions.
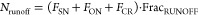
8
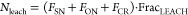
9

Figure 1 shows the general methodology for calculating nitrogen flows throughout the network and the respective data sources. It shows that the flows are calculated for each one of the four main stages: (1) feed, (2) animal operations, (3) processing, and (4) consumption where each stage is dependent on the previous.

To estimate nitrogen losses during processing, we assume that nitrogen is retained in the form of amino acids which are combined to form protein throughout the body in muscles, bone, skin, etc. Since fat is made up of glycerol and fatty acids, it is assumed to not contain any nitrogen. Nitrogen is lost to wastewater at the rendering plant during processing where the protein bonds are broken down and nitrogen is released.^57^ The dressing percentage is assumed to be approximately 63%.^58^ The dressing percentage is the fraction of the live animal that comprises the hot carcass, which is the weight of the carcass without the head, hide, horns, and gut fill. Assuming nitrogen is stored throughout the body evenly minus the fat, between the live animal and the obtaining of the carcass, approximately 37% of the retained nitrogen is lost in the inedible parts. Approximately 15% of the hot carcass is made up of bones.^59^ As such, of the remaining 63% nitrogen, approximately 48% of nitrogen is in the edible tissue.

Once the nitrogen intensities are obtained, we computed the nitrogen lost in each phase and the embodied nitrogen that proceeds to the next node of the beef supply chain. We assumed that the products are perfectly mixed at each stage and the links follow the same proportion as the origin node’s incoming links. For example, if a county received meat from one slaughterhouse that buys cattle from two feedlots in equal proportion, then the county of beef consumption is also linked to both feedlots in the same proportion. This nitrogen network is used to estimate consumption-based metrics.

## Results

### Feed Network

A total of 51 megatonne (Mt) of feed was moved toward animal operations, with 55% going toward feedlots and 45% toward ranches, not including pasture intake which accounts for the majority of dry matter intake during the cow-calf phase. This included flows of corn grain (12.8 Mt), corn silage (7.5 Mt), alfalfa (3.3 Mt), and distiller’s grains (4.5 Mt) toward feedlots and corn grain (4.4 Mt), hay (18 Mt), and soybeans (0.28 Mt) toward ranches. Moreover, 48.5% of all of the feed movements were either corn grain or corn silage, 42% was either alfalfa or other hay, 8% was distiller’s grain, and 0.5% was soybeans. On average, 8 kg of animal feed was consumed to produce 1 kg of boneless beef.

**Figure 2:**
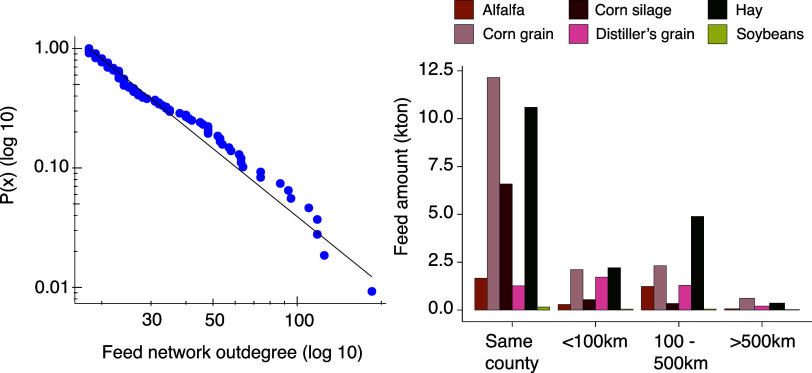
(a) Out-degree distribution of nodes in the feed network. The theoretical power-law cumulative distribution function is represented by a straight line, and the empirical distribution is represented by dots. (b) Amount of animal feed sourced by distance between county centroids.

A total of 2674 counties are in the feed network, with 86% being both a feed and animal producer, 9% being feed producer only, and 5% being animal producer only. Real-world networks often present highly skewed degree distributions, with very few nodes that are highly connected. Figure 2a shows that the feed network does present this characteristic with the majority of counties having a small number of outgoing connections. Scale-free networks have degree distributions that are characterized by a power law with probability proportional to *x*^–α^ with the α parameter often falling between 2 and 3.^60^Figure 2a shows the empirical cumulative distribution of out-degree in the feed network. The empirical probability was proportional to *x*^–2.88^, and the Kolmogorov–Smirnov test supports that power law distribution is a plausible explanation (*p*-value = 0.42). Similarly, the network link distribution can also be reasonably explained with a power law (*p*-value = 0.60) with α parameter of 2.6. Therefore, the feed network is characterized by most nodes having fewer connections and the majority of links having low values.

The network also enables an understanding of how far the feed-producing counties are from the counties with animal operations. Figure 2b shows that most links in the network were self-loops; i.e., the origin and destination were the same county. Overall, most feed (75%) was obtained from a crop producer within an 85 km radius. This finding is in line with values observed in a survey with cattle producers in the U.S.,^61^ where the median of the distance between feed producer and animal operation was 30 km with a maximum of 3000 km and the 75th percentile was 84 km, while our results show a median of 50 km and a maximum of 2000 km. It is to be noted that the optimization model in our work does not quantify distance within county shipments, and estimates are based on average distances between two counties. Feeds such as corn grain and corn silage are more likely to be purchased within the same county, as 76% of corn grain flows were self-loops. On the other hand, the model showed that distiller’s grains and soybeans were generally sourced from counties that were further away from animal operations with averages of 283 and 190 km, respectively.

### Nitrogen Flows

**Figure 3:**
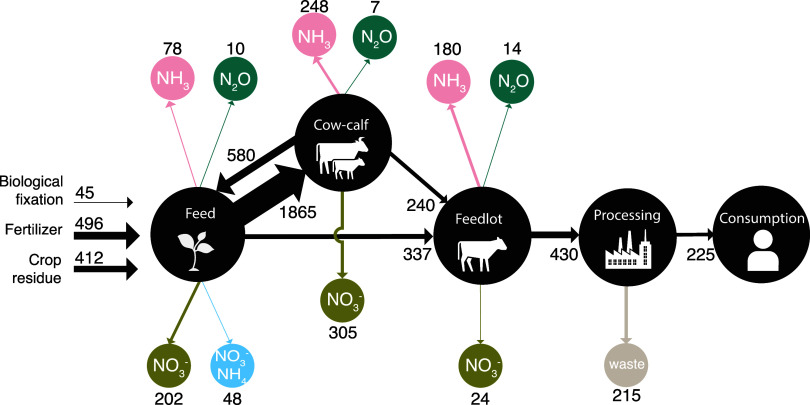
Association of nitrogen flows with beef supply chains. Nitrogen retention and losses in animal feed, ranch (cow-calf), feedlot (backgrounding and finishing), processing, and consumption phases in Gg N for 1 year’s worth of beef consumption in the United States are shown. All flows are for the year 2017.

Figure 3 shows that approximately 1530 gigagrams N (Gg N) were mobilized to produce fed cattle beef consumed in 2017, out of which only 225 Gg N ended up in the product itself in the form of tissue. This translates to an overall efficiency of approximately 15%. However, out of all inputs, 640 Gg of N is considered new nitrogen (biological fixation, synthetic fertilizers), with the rest coming from crop residues and recycled manure. Thus, the partial factor productivity, which is defined as the ratio of nitrogen that ended up in the product to the new nitrogen input, was 35%. The virtual nitrogen factor (VNF) defined in Leach et al.^20^ captures how much nitrogen was lost to the environment per unit of nitrogen in the product. They reported a VNF of 8.5 for beef, whereas we observed a VNF of 5.9 for the modeled beef network. Differences can be due to temporal scale, assumptions in feed requirements and production systems, and calculation methods. They obtained the VFN with estimated factors that reflect the percentage of nitrogen in the chain in comparison to the amount lost to the environment, while we calculated nitrogen losses with IPCC Tier 1 and Tier 2 methodologies and energy required for animal growth. Since large portions of losses occur during feed, feed ratio and productivity are also important variables; overall productivity has increased in the last decades. Nonetheless, both VFN values reflect that beef has a relatively high nitrogen footprint and can be used to inform consumption-based accounting of nitrogen.

Approximately 1330 Gg N was released into the environment due to beef consumption in 2017. Most nitrogen was lost in the form of nitrate (NO_3_^–^) through leaching into the soil (40%), followed by ammonia (NH_3_) through volatilization (39%), waste or wastewater during processing (15%), runoff (3%), and direct nitrous oxide (N_2_O) (2%) emissions. Approximately 25% of nitrogen losses occurred during feed production, 60% during animal operations, and 15% during processing. Pasture nitrogen management is particularly important because it is responsible for the bulk of nitrogen releases mainly through leaching. Therefore, cow-calf operations, due to a longer time in pasture and high feed requirements, had an associated nitrogen loss from both feed and animal operations of 751 Gg N, much higher than 363 Gg N resulting from feedlot operations. Combined, nitrogen emissions at the feedlot gate were approximately 1.11 teragrams (Tg) N, and is in good agreement with the reactive nitrogen emissions estimated by Rotz et al.^37^ of 1.47 Tg N for beef systems in the U.S.

Statistically speaking, the county-level nitrogen emissions can be reasonably described with a power-law distribution (*p*-value >0.1). This indicates that a few counties receive higher nitrogen loads while most counties have relatively low nitrogen emissions. Emissions in 100 counties sum up to 40% of nitrogen emissions in the network. The county with the highest nitrogen losses was Ford (KS) with 24 Gg N, where most of it is due to the packing plant (85%) located within its boundaries. Most counties within the top 10 in the nitrogen losses ranking had most (>50%) of their losses occurring in the processing stage. Exceptions include Deaf Smith (TX) with 13 Gg N losses, mostly due to cow-calf operations (49%) and feedlots (46%), and Haskell (KS) with 12 Gg N from cow-calf (32%) and feedlot (65%). Nitrogen losses in the top 10 counties sum up to 10% of national nitrogen losses associated with beef. These results also highlight that although processing nitrogen emissions make up a smaller share at the national level (15%), it is important to evaluate these systems at a smaller scale.

### Production vs Consumption-Based Accounting

**Figure 4:**
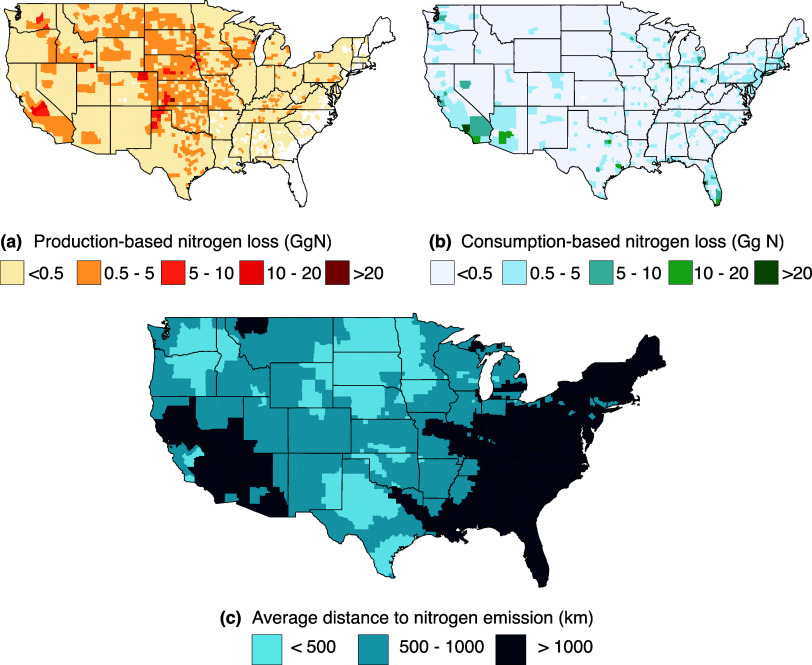
County-level nitrogen losses from (a) production-based accounting and (b) consumption-based accounting. The weighted average distance between consumption and nitrogen losses during production is depicted in map (c).

Figure 4 shows a comparison of the production- and consumption-based nitrogen emissions at the county level. Consumption in 100 counties is associated with 42% of total nitrogen losses. Highly populated areas such as Los Angeles (CA), Cook (IL), Harris (TX), New York City counties (NY), and Maricopa (AZ) have associated nitrogen losses between 16 and 34 Gg N for 1 year of beef consumption. On average across the network, consumption in a single county is associated with nitrogen losses occurring in 240 counties. The average distance between the location where nitrogen is released and the location where beef is consumed is approximately 975 km. The maximum distance observed was 5000 km between Florida and Washington.

Per capita county-level consumption-based nitrogen footprints ranged from 3.2 to 4.6 kg N/capita with an average of 4.0 kg N/capita, comparable to 5.6 kg N/capita reported by Liang et al.^24^ Reactive nitrogen loss per carcass weight from Rotz et al.^37^ ranged from 112 to 272 g N/kg CW while our estimates range between 111 and 160 g N/kg CW with an average of 135 g N/kg CW. The nitrogen footprint in terms of boneless beef consumed ranged from 168 g N/kg beef to 239 kg N/kg beef, with an average of 205 kg N/kg beef.

**Figure 5:**
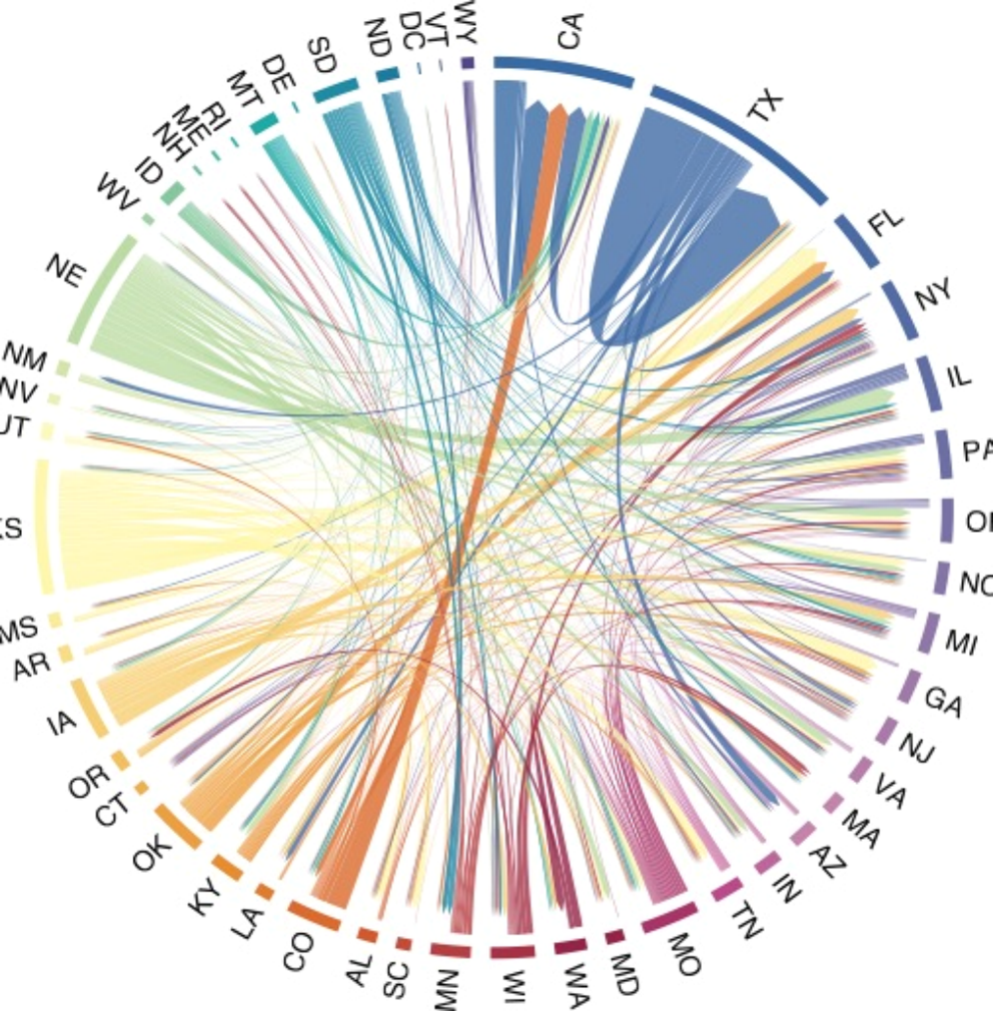
Chord diagram of nitrogen flows between states that represent the amount of nitrogen released at the origin state associated with the amount of beef consumed at the destination state. The states are ordered by the sum of inflows starting at the 90-degree mark. Inflows are identified by arrows and larger gap to arc.

Figure 5 reveals that most nitrogen emissions from beef production occur in a state different from where the beef is consumed, with substantial outflows from agricultural states and inflows to populous states. The chord diagram is organized from the largest to smallest total inflows starting with California at the 90-degree mark, followed by Texas, Florida, New York, and Pennsylvania which form the set of highest inflows. The two largest flow values are self-loops in Texas (89 Gg N) and California (36 Gg N), followed by links from Kansas to Florida (29 Gg N), from Colorado to California (29 Gg N), from Texas to California (27 Gg N), and Nebraska to Illinois (26 Gg N). Nebraska, South Dakota, Kansas, North Dakota, and Wyoming have the largest outflow-to-inflow ratios, whereas states in New England generally have the highest inflow-to-outflow ratios. Approximately 15% of the link values are self-loops; therefore, most nitrogen emissions occur in a state other than where the beef was consumed. Figure S3 in the Supporting Information maps the relationship between inflows and outflows of nitrogen.

### Sensitivity Analysis

Sensitivity analysis was used to evaluate how the total nitrogen loss and footprint metrics change based on modeling assumptions. Since data on manure application on pasture are not available spatially, we did not assume that manure was being added in addition to the manure deposited by the cattle during grazing. However, mechanical manure application may occur, depending on specific nutrient levels. In addition, environmental protection agencies at the state level in the United States and university extension programs often provide guidelines and regulations for the application of manure in different contexts.^62,63^ The implication of using manure on pastures for forage yield and nutrient balances is an active area of research. Wilson et al.^64^ showed that liquid hog manure at 57 kg N/acre in addition to deposited manure on pastures helped improve carrying capacity and forage quality. Kingery et al.^65^ argue that poultry manure application between 87 and 290 kg N/acre can be a suitable manure management practice, but long-term excessive levels of manure application on pasture can have adverse effects on surface integrity and animal productivity. Van der Weerden et al.^66^ investigated manure application rates between 13 and 46 kg N/acre for nitrogen emissions in New Zealand pastures. A global assessment reported by Potter et al.^67^ showed that manure is generally applied at rates greater than 14 kg N/acre where applications occur. For our sensitivity analysis, we have considered manure treatments of 14 and 55 kg N/acre on pasture during the cow-calf phase in addition to the nitrogen deposited during grazing which averaged 17 kg N/acre according to calculated N excretion and stocking rates. The impact of manure application on total nitrogen balance and emissions is shown in Table 1.

**Table 1:** Sensitivity Analysis of the Total Nitrogen Inputs and Losses as a Result of Changes in Manure Application and Crop Removal Fraction Changes^a^

Scenario	Variable	Value	Unit	Nitrogen input	Nitrogen loss
Baseline	Manure_applied_	0	kg N/acre	1532	1330
	Frac_removed_	0	%		
					
	Manure_applied_	14	kg N/acre	2209	1467
	Manure_applied_	55	kg N/acre	4280	2023
	Frac_removed_	50	%	1452	1310
	Frac_removed_	90	%	1391	1224
	Manure_recycled_	14	kg N/acre	1588	1353
	Manure_recycled_	55	kg N/acre	1753	1548
	Manure_recycled_	100	kg N/acre	1932	1761

a Nitrogen input includes recycling into feed system.

Another factor that influences the nitrogen balance is crop residues. We adopted the default value of crop residue removal fraction (Frac_removed_) of zero according to IPCC guidelines for the case where data is not available. However, in addition to the grass being grazed by the animals, grass may be removed for other feeding purposes, such as drying and feeding as hay. Thus, we conducted sensitivity analysis with Frac_removed_ for pasture at 50 and 90% removed.

To better understand the potential of manure recycling, we also ran a sensitivity analysis for the application of manure produced in feedlots on cropland used to produce feed, namely, corn grain, corn silage, and alfalfa. We have tested the same rates as the manure application on pastureland: 14 and 55 kg N/acre as well as 100 kg N/acre. The latter was added to be comparable to current rates of synthetic fertilizer application, which can reach 150 kg N/acre. We found that to reach a 100 kg N/acre application rate from manure into the main feed crops necessary for feedlots, 400 Gg N would be required. This is approximately 58% of the total nitrogen excreted by backgrounding and finishing cattle (687 Gg N), revealing the potential for reducing the reliance on new nitrogen. For more details on nitrogen flows of these scenarios, see Figures S3 and S4, Supporting Information.

The rate of nitrogen from manure application significantly affects nitrogen balance and nitrogen loss. These two factors can change total nitrogen loss from 1330 Gg N to 1224 when the nitrogen from crop residues is reduced or as high as 2023 when there is significant nitrogen from manure. In the absence of available data, the model does not consider changes in forage yield and quality after receiving additional nitrogen from manure application and assumes the entirety of pasturelands receiving additional nitrogen from mechanical manure application. Nitrogen requirements must be evaluated according to soil conditions, weather, and cost. These values serve as a reference for the uncertainty in our estimates.

## Discussion and Outlook

This study aimed to fill a knowledge gap pertaining to spatially explicit nitrogen flows associated with beef supply chains in the United States. Using information about feed production and feed requirements by animals, we developed a county-level network of feed flows. We showed that a network constructed based on the optimization of transportation costs resulted in most feed being sourced from within the same county. This reveals the potential for increasing circularity through recycling of manure as nitrogen source for feed crops, in line with the concept of “manuresheds” as defined by Spiegal et al.^68^ as the “land surrounding animal feeding operations onto which manure nutrients can be redistributed”. The network framework developed in this study can aid in devising strategies to improve nitrogen recycling in conjunction with a national effort involving scientists, and policy- and decision-makers to ensure that food systems remain within the carrying capacity of the surrounding ecosystems.^4,5^

On average, distances between feed producer and animal operations were similar to previously reported values from surveys of producers. We sought to utilize the best data available and the surveys by Asem-Hiablie et al.^33−36^ provided comprehensive regional information about cattle production practices and enabled us to track feed movements for major feeds according to the phase and location of animal operations. Nonetheless, the optimized flows might not necessarily represent actual flows, and this approach has intrinsic limitations. In the absence of animal-specific data on the availability of feed crops, the feed availability within a county was estimated based on general allocation values for food, fuel, and feed. However, there are other livestock categories that require the same feed crops, leading to competition for other uses. Thus, the feed availability within a county might be overestimated. Future work can build upon the optimization framework presented in this study and account for feed flows to other livestock supply chains by enabling decentralized decision-making.

Another limitation is the supply chain modeling of the final steps of beef. The system boundary for the study was limited to feed, ranches, feedlots, slaughterhouses, and consumption, the data for which was publicly available. As such, it was beyond the scope of this study to include distribution centers or retail in the network. Consequently, the network in this work might not be comparable directly with freight data such as freight analysis framework (FAF) or commodity flow survey (CFS) because the origin points are naturally different, a slaughterhouse instead of distribution centers, for example. In addition, the optimization framework might not capture the diversity of branded products in a county. As we optimize for transportation and purchasing costs, the network nodes will be attached preferentially to the nearest supplier according to the amount being hauled. Future work can model the beef network via dynamic models with market and socioeconomic constraints to obtain a more realistic network.

The nitrogen losses and retention network in our work were assessed with the best available information on nitrogen flows. However, some nitrogen values were limiting. For example, we used spatially explicit values for volatilization fractions, but spatially explicit values were not available for runoff and leaching. We calculated runoff fractions according to published work based on elevation maps, but general leaching fractions based on IPCC reports were considered with the assumption that runoff and leaching together represent 24% of losses. Thus, leaching was assumed to be a function of the runoff. More research is needed to estimate spatially explicit leaching fractions. Emission factors were also assumed to be homogeneous in space.

Our work aids in an understanding of the relationship between consumption and environmental impacts at origin. The use of an optimization-based framework to model supply chain linkages is an efficient way to generate data based on available information on capacity, demand, purchase price, and transportation costs. It is also an appropriate resource for discussions around supply chain transparency and sustainability, as we aim to understand how and where consumption patterns affect the environment.

## Supporting Information Available

Additional data, results, and methodological details (PDF)
Descriptions of cattle management, fertilizer usage, and nitrogen losses, as well as additional sensitivity analysis results (XLSX)

## References

[ref1] Canfield D. E.; Glazer A. N.; Falkowski P. G. The Evolution and Future of Earth’s Nitrogen Cycle. Science 2010, 330 (6001), 192–196. 10.1126/science.1186120. 20929768

[ref2] Bodirsky B. L.; Popp A.; Lotze-Campen H.; Dietrich J. P.; Rolinski S.; Weindl I.; Schmitz C.; Müller C.; Bonsch M.; Humpenöder F.; Biewald A.; Stevanovic M. Reactive Nitrogen Requirements to Feed the World in 2050 and Potential to Mitigate Nitrogen Pollution. Nat. Commun. 2014, 5 (1), 3858 10.1038/ncomms4858. 24819889

[ref3] Galloway J. N.; Schlesinger W. H.; Levy H.; Michaels A.; Schnoor J. L. Nitrogen Fixation: Anthropogenic Enhancement-Environmental Response. Global Biogeochem. Cycles 1995, 9 (2), 235–252. 10.1029/95GB00158.

[ref4] Rockström J.; Steffen W.; Noone K.; Persson Å.; Chapin F. S.; Lambin E. F.; Lenton T. M.; Scheffer M.; Folke C.; Schellnhuber H. J.; Nykvist B.; De Wit C. A.; Hughes T.; Van Der Leeuw S.; Rodhe H.; Sörlin S.; Snyder P. K.; Costanza R.; Svedin U.; Falkenmark M.; Karlberg L.; Corell R. W.; Fabry V. J.; Hansen J.; Walker B.; Liverman D.; Richardson K.; Crutzen P.; Foley J. A. A Safe Operating Space for Humanity. Nature 2009, 461 (7263), 472–475. 10.1038/461472a. 19779433

[ref5] Richardson K.; Steffen W.; Lucht W.; Bendtsen J.; Cornell S. E.; Donges J. F.; Drüke M.; Fetzer I.; Bala G.; von Bloh W.; Feulner G.; Fiedler S.; Gerten D.; Gleeson T.; Hofmann M.; Huiskamp W.; Kummu M.; Mohan C.; Nogués-Bravo D.; Petri S.; Porkka M.; Rahmstorf S.; Schaphoff S.; Thonicke K.; Tobian A.; Virkki V.; Wang-Erlandsson L.; Weber L.; Rockström J. Earth beyond Six of Nine Planetary Boundaries. Sci. Adv. 2023, 9 (37), eadh2458 10.1126/sciadv.adh2458. 37703365 PMC10499318

[ref6] Singh S.; Bakshi B. R. Accounting for the Biogeochemical Cycle of Nitrogen in Input-Output Life Cycle Assessment. Environ. Sci. Technol. 2013, 47 (16), 9388 10.1021/es4009757. 23869533

[ref7] Sobota D. J.; Compton J. E.; McCrackin M. L.; Singh S. Cost of Reactive Nitrogen Release from Human Activities to the Environment in the United States. Environ. Res. Lett. 2015, 10 (2), 025006 10.1088/1748-9326/10/2/025006.

[ref8] Galloway J. N.; Aber J. D.; Erisman J. W.; Seitzinger S. P.; Howarth R. W.; Cowling E. B.; Cosby B. J. The Nitrogen Cascade. BioScience 2003, 341–356. 10.1641/0006-3568(2003)053[0341:TNC]2.0.CO;2.

[ref9] Erisman J. W.; Galloway J. N.; Seitzinger S.; Bleeker A.; Dise N. B.; Roxana Petrescu A. M.; Leach A. M.; de Vries W. Consequences of Human Modification of the Global Nitrogen Cycle. Philos. Trans. R. Soc., B 2013, 368 (1621), 20130116 10.1098/rstb.2013.0116. PMC3682738 23713116

[ref10] Lassaletta L.; Billen G.; Grizzetti B.; Garnier J.; Leach A. M.; Galloway J. N. Food and Feed Trade as a Driver in the Global Nitrogen Cycle: 50-Year Trends. Biogeochemistry 2014, 118 (1–3), 225–241. 10.1007/s10533-013-9923-4.

[ref11] Leach A. M.; Emery K. A.; Gephart J.; Davis K. F.; Erisman J. W.; Leip A.; Pace M. L.; D’Odorico P.; Carr J.; Noll L. C.; Castner E.; Galloway J. N. Environmental Impact Food Labels Combining Carbon, Nitrogen, and Water Footprints. Food Policy 2016, 61, 213 10.1016/j.foodpol.2016.03.006.

[ref12] Lassaletta L.; Billen G.; Romero E.; Garnier J.; Aguilera E. How Changes in Diet and Trade Patterns Have Shaped the N Cycle at the National Scale: Spain (1961–2009). Reg. Environ. Change 2014, 14 (2), 785–797. 10.1007/s10113-013-0536-1.

[ref13] Steinfeld H.; Gerber P.; Wassenaar T. Livestock’s Long Shadow: Environmental Issues and Options; United Nations Food and Agriculture Organization, 2006.

[ref14] Hutchins C. Feed Grains Sector at a Glance. USD Sector at Glace; USDA ERS, 2023.

[ref15] Uwizeye A. Nutrient Challenges in Global Livestock Supply Chains : An Assessment of Nitrogen Use and Flows. Ph.D. Thesis, Wageningen University: Wageningen, 2019.

[ref16] Silver W. S. Biological Nitrogen Fixation. Science 1967, 157 (784), 100–102. 10.1126/science.157.3784.100. 6067409

[ref17] Genzebu D.; Weldeslasse G. T. The Role of Bacteria in Nitrogen Metabolism in the Rumen with Emphasis of Cattle. Res. J. Agric. Environ. Manage. 2015, 4 (7), 282–290.

[ref18] Smil V. Eating Meat: Evolution, Patterns, and Consequences. Popul. Dev. Rev. 2002, 28 (4), 599 10.1111/j.1728-4457.2002.00599.x.

[ref19] Flynn K. C.; Spiegal S.; Kleinman P. J. A.; Meinen R. J.; Smith D. R. Manureshed Management to Overcome Longstanding Nutrient Imbalances in US Agriculture. Resour., Conserv. Recycl. 2023, 188, 106632 10.1016/j.resconrec.2022.106632.

[ref20] Leach A. M.; Galloway J. N.; Bleeker A.; Erisman J. W.; Kohn R.; Kitzes J. A Nitrogen Footprint Model to Help Consumers Understand Their Role in Nitrogen Losses to the Environment. Environ. Dev. 2012, 1 (1), 40–66. 10.1016/j.envdev.2011.12.005.

[ref21] Pierer M.; Winiwarter W.; Leach A. M.; Galloway J. N. The Nitrogen Footprint of Food Products and General Consumption Patterns in Austria. Food Policy 2014, 49 (P1), 128–136. 10.1016/j.foodpol.2014.07.004.

[ref22] Shibata H.; Cattaneo L. R.; Leach A. M.; Galloway J. N. First Approach to the Japanese Nitrogen Footprint Model to Predict the Loss of Nitrogen to the Environment. Environ. Res. Lett. 2014, 9 (11), 115013 10.1088/1748-9326/9/11/115013.

[ref23] Leip A.; Weiss F.; Lesschen J. P.; Westhoek H. The Nitrogen Footprint of Food Products in the European Union. J. Agric. Sci. 2014, 152, S20–S33. 10.1017/S0021859613000786.

[ref24] Liang X.; Leach A. M.; Galloway J. N.; Gu B.; Lam S. K.; Chen D. Beef and Coal Are Key Drivers of Australia’s High Nitrogen Footprint. Sci. Rep. 2016, 6, 39644 10.1038/srep39644. 28008979 PMC5180353

[ref25] Cattell Noll L.; Leach A. M.; Seufert V.; Galloway J. N.; Atwell B.; Erisman J. W.; Shade J. The Nitrogen Footprint of Organic Food in the United States. Environ. Res. Lett. 2020, 15 (4), 045004 10.1088/1748-9326/ab7029.

[ref26] Algren M.; Landis A. E.; Costello C. Estimating Virtual Nitrogen Inputs to Integrated u.s. Corn Ethanol and Animal Food Systems. Environ. Sci. Technol. 2021, 55 (12), 8393–8400. 10.1021/acs.est.1c02208. 34077190

[ref27] Zhang X.; Zou T.; Lassaletta L.; Mueller N. D.; Tubiello F. N.; Lisk M. D.; Lu C.; Conant R. T.; Dorich C. D.; Gerber J.; Tian H.; Bruulsema T.; Maaz T. M. C.; Nishina K.; Bodirsky B. L.; Popp A.; Bouwman L.; Beusen A.; Chang J.; Havlík P.; Leclère D.; Canadell J. G.; Jackson R. B.; Heffer P.; Wanner N.; Zhang W.; Davidson E. A. Quantification of Global and National Nitrogen Budgets for Crop Production. Nat. Food 2021, 2 (7), 529–540. 10.1038/s43016-021-00318-5. 37117677

[ref28] Zhang X.; Ren C.; Gu B.; Chen D. Uncertainty of Nitrogen Budget in China. Environ. Pollut. 2021, 286, 117216 10.1016/j.envpol.2021.117216. 33965801

[ref29] Uwizeye A.; de Boer I. J. M.; Opio C. I.; Schulte R. P. O.; Falcucci A.; Tempio G.; Teillard F.; Casu F.; Rulli M.; Galloway J. N.; Leip A.; Erisman J. W.; Robinson T. P.; Steinfeld H.; Gerber P. J. Nitrogen Emissions along Global Livestock Supply Chains. Nat. Food 2020, 1 (7), 437–446. 10.1038/s43016-020-0113-y.

[ref30] McLellan E. L.; Cassman K. G.; Eagle A. J.; Woodbury P. B.; Sela S.; Tonitto C.; Marjerison R. D.; Van Es H. M. The Nitrogen Balancing Act: Tracking the Environmental Performance of Food Production. BioScience 2018, 68 (3), 194 10.1093/biosci/bix164. 29662247 PMC5894078

[ref31] Ostroski A.; Lagos T.; Prokopyev O. A.; Khanna V. Consumption-Based Accounting for Tracing Virtual Water Flows Associated with Beef Supply Chains in the United States. Environ. Sci. Technol. 2022, 56, 16347–16356. 10.1021/acs.est.2c03986. 36283089

[ref32] Statistics Canada—Agriculture Division. Livestock Feed Requirements Study (23-501-X), 2003. http://www5.statcan.gc.ca/olc-cel/olc.action?objId=23-501-X&objType=2&lang=en&limit=0 (accessed July 16, 2020).

[ref33] Asem-Hiablie S.; Alan Rotz C.; Stout R.; Dillon J.; Stackhouse-Lawson K. Management Characteristics of Cow-Calf, Stocker, and Finishing Operations in Kansas, Oklahoma, and Texas. Prof. Anim. Sci. 2015, 31 (1), 1–10. 10.15232/pas.2014-01350.

[ref34] Asem-Hiablie S.; Rotz C. A.; Stout R.; Stackhouse-Lawson K. Management Characteristics of Beef Cattle Production in the Northern Plains and Midwest Regions of the United States. Prof. Anim. Sci. 2016, 32 (6), 736–749. 10.15232/pas.2016-01539.

[ref35] Asem-Hiablie S.; Rotz C. A.; Stout R.; Fisher K. Management Characteristics of Beef Cattle Production in the Western United States. Prof. Anim. Sci. 2017, 33 (4), 461–471. 10.15232/pas.2017-01618.

[ref36] Asem-Hiablie S.; Rotz C. A.; Stout R.; Place S. Management Characteristics of Beef Cattle Production in the Eastern United States. Prof. Anim. Sci. 2018, 34 (4), 311–325. 10.15232/pas.2018-01728.

[ref37] Rotz C. A.; Asem-Hiablie S.; Place S.; Thoma G. Environmental Footprints of Beef Cattle Production in the United States. Agric. Syst. 2019, 169, 1–13. 10.1016/j.agsy.2018.11.005.

[ref38] Mueller N. D.; Gerber J. S.; Johnston M.; Ray D. K.; Ramankutty N.; Foley J. A. Closing Yield Gaps through Nutrient and Water Management. Nature 2012, 490 (7419), 254–257. 10.1038/nature11420. 22932270

[ref39] West P. C.; Gerber J. S.; Engstrom P. M.; Mueller N. D.; Brauman K. A.; Carlson K. M.; Cassidy E. S.; Johnston M.; MacDonald G. K.; Ray D. K.; Siebert S. Leverage Points for Improving Global Food Security and the Environment. Science 2014, 345 (6194), 325–328. 10.1126/science.1246067. 25035492

[ref40] Gonzales D.; Searcy E. M.; Ekşioĝlu S. D. Cost Analysis for High-Volume and Long-Haul Transportation of Densified Biomass Feedstock. Transp. Res. Part A: Policy Pract. 2013, 49, 48–61. 10.1016/j.tra.2013.01.005.

[ref41] Robusto C. C. The Cosine-Haversine Formula. Am. Math. Mon. 1957, 64 (1), 38 10.2307/2309088.

[ref42] Gastelle J. Transportation of U.S. Grains: A Modal Share Analysis; U.S. Dept. of Agriculture, Agricultural Marketing Service, 2017. 10.9752/TS049.10-2017.

[ref43] Dooley F. J.; Martens B. J. Transportation and Logistics in Distiller’s Grain Markets. In Using Distillers Grains in the U.S. and International Livestock and Poultry Industries; Babcock B. A.; Hayes D. J.; Lawrence J. D., Eds.; Midwest Agribusiness Trade Research and Information Center, 2008.

[ref44] Bertsimas D.; Tsitsiklis J. Introduction to Linear Optimization, Athena Scientific Series in Optimization and Neural Computation; Athena Scientific, 1997.

[ref45] Lovelock C. E.; Evans C.; Barros N.; Prairie Y.; Alm J.; Bastviken D.; Beaulieu J. J.; Garneau M.; Harby A.; Harrison J.; Pare D.; Raadal H. L.; Sherman B.; Zhang C.; Ogle S. M.; Grinham A.; Deemer B.; Aurelio dos Santos M.; Kosten S.; Peacock M.; Li Z.; Stepanenko V. Wetlands. In 2019 Refinement to the 2006 IPCC Guidelines for National Greenhouse Gas Inventories. Vol. 4: Agricultura, Forestry and Other Land Use (AFOLU); Calvo Buendia E.; Tanabe K.; Kranjc A.; Baasansuren J.; Fukuda M.; Ngarize S.; Osako A.; Pyronshenko Y.; Shermanau P.; Frederici S., Eds.; IPCC: Switzerland, 2019; Chapter 7, Vol. 4.

[ref46] USDA. National Agricultural Statistics Service—Surveys—Agricultural Chemical Use Program, 2022. https://www.nass.usda.gov/Surveys/Guide_to_NASS_Surveys/Chemical_Use/ (accessed July 06, 2022).

[ref47] Crop Nutrient Tool|USDA PLANTS, 2022. https://plantsorig.sc.egov.usda.gov/npk/main (accessed July 06, 2022).

[ref48] Ciampitti I. A.; Salvagiotti F. Soybeans and Biological Nitrogen Fixation: A Review. Better Crops Plant Food 2018, 102 (3), 5–7. 10.24047/BC10235.

[ref49] Issah G.; Schoenau J. J.; Lardner H. A.; Diane Knight J. Nitrogen Fixation and Resource Partitioning in Alfalfa (*Medicago sativa* L.), Cicer Milkvetch (*Astragalus cicer* L.) and Sainfoin (*Onobrychis viciifolia* Scop.) Using 15N Enrichment under Controlled Environment Conditions. Agronomy 2020, 10 (9), 1438 10.3390/AGRONOMY10091438.

[ref50] Eirckson G.; Milton T. Nitrogen and P Excretion in Feedlot Cattle and Its Fate. Using Dietary Strategies to Reduce the Nutrient Excretion of Feedlot Cattle; Livestock and Poultry Environmental Learning Community, National Institute of Food and Agriculture, National Cooperative Extension Resource, 2019.

[ref51] IPCC. Volume 4: Agriculture, Forestry and Other Land Use. Chapter 11: N_2_O Emissions From Managed Soils, and CO_2_ Emissions from Lime and Urea Application. 2019 Refinement to the 2006 IPCC Guidelines for National Greenhouse Gas Inventories; IPCC, 2019.

[ref52] IPCC. Volume 4: Agriculture, Forestry and Other Land Use. Chapter 10: Emissions Form Livestock and Manure Management. 2019 Refinement to the 2006 IPCC Guidelines for National Greenhouse Gas Inventories; IPCC, 2019.

[ref53] Vira J.; Hess P.; Melkonian J.; Wieder W. R. An Improved Mechanistic Model for Ammonia Volatilization in Earth System Models: Flow of Agricultural Nitrogen Version 2 (FANv2). Geosci. Model Dev. 2020, 13 (9), 4459–4490. 10.5194/gmd-13-4459-2020.

[ref54] U.S. EPA. National Emission Inventory—Ammonia Emissions from Animal Agricultural Operations, 2006. ftp://ftp.epa.gov/EmisInventory/2002finalnei/documentation/nonpoint/2002nei%5C_final%5C_nonpoint%5C_documentation0206version.pdf.

[ref55] Velthof G. L.; Oudendag D.; Witzke H. P.; Asman W. A. H.; Klimont Z.; Oenema O. Integrated Assessment of Nitrogen Losses from Agriculture in EU-27 Using MITERRA-EUROPE. J. Environ. Qual. 2009, 38 (2), 402–417. 10.2134/jeq2008.0108. 19202011

[ref56] FAO. Global Livestock Environmental Assessment Model; FAO, 2018. 10.1017/S1751731117001847 28789724

[ref57] Brennan B.; Lawler J.; Regan F. Recovery of Viable Ammonia-Nitrogen Products from Agricultural Slaughterhouse Wastewater by Membrane Contactors: A Review. Environ. Sci. 2021, 7 (2), 259–273. 10.1039/D0EW00960A.

[ref58] Holland R.; Loveday D.; Ferguson K. How Much Meat to Expect from a Beef Carcass, 2014. https://ag.tennessee.edu (accessed Feb 09, 2020).

[ref59] South Dakota State University. How Much Meat Can You Expect from a Fed Steer? iGrow. http://igrow.org/livestock/beef/how-much-meat-can-you-expect-from-a-fed-steer/.

[ref60] Broido A. D.; Clauset A. Scale-Free Networks Are Rare. Nat. Commun. 2019, 10 (1), 1017 10.1038/s41467-019-08746-5. 30833554 PMC6399239

[ref61] Beam A. L.; Thilmany D. D.; Pritchard R. W.; Garber L. P.; van Metre D. C.; Olea-Popelka F. J. Distance to Slaughter, Markets and Feed Sources Used by Small-Scale Food Animal Operations in the United States. Renewable Agric. Food Syst. 2016, 31 (1), 49–59. 10.1017/S1742170514000441.

[ref62] Department of Environmental Protection; Commonwealth of Pennsylvania. Land Application of Manure; Department of Environmental Protection; Commonwealth of Pennsylvania, 2010; Vol 31.

[ref63] James R.; van Houtert M. F. J.; Elder K.; Lyon W. F. Ohio Livestock Manure Management Guide; Ohio State University, 2006.

[ref64] Wilson C.; Undi M.; Tenuta M.; Wittenberg K. M.; Flaten D.; Krause D. O.; Entz M. H.; Holley R.; Ominski K. H. Pasture Productivity, Cattle Productivity and Metabolic Status Following Fertilization of a Grassland with Liquid Hog Manure: A Three-Year Study. Can. J. Anim. Sci. 2010, 90 (2), 233 10.4141/CJAS09037.

[ref65] Kingery W. L.; Wood C. W.; Delaney D. P.; Williams J. C.; Mullins G. L.; van Santen E. Implications of Long-Term Land Application of Poultry Litter on Tall Fescue Pastures. J. Prod. Agric. 1993, 6 (3), 390 10.2134/jpa1993.0390.

[ref66] Van Der Weerden T. J.; Luo J.; Dexter M.; Rutherford A. J. Nitrous Oxide, Ammonia and Methane Emissions from Dairy Cow Manure during Storage and after Application to Pasture. N. Z. J. Agric. Res. 2014, 57 (4), 354–369. 10.1080/00288233.2014.935447.

[ref67] Potter P.; Ramankutty N.; Bennett E. M.; Donner S. D. Characterizing the Spatial Patterns of Global Fertilizer Application and Manure Production. Earth Interact. 2010, 14 (2), 1–22. 10.1175/2009EI288.1.

[ref68] Spiegal S.; Kleinman P. J. A.; Endale D. M.; Bryant R. B.; Dell C.; Goslee S.; Meinen R. J.; Flynn K. C.; Baker J. M.; Browning D. M.; McCarty G.; Bittman S.; Carter J.; Cavigelli M.; Duncan E.; Gowda P.; Li X.; Ponce-Campos G. E.; Cibin R.; Silveira M. L.; Smith D. R.; Arthur D. K.; Yang Q. Manuresheds: Advancing Nutrient Recycling in US Agriculture. Agric. Syst. 2020, 182, 102813 10.1016/j.agsy.2020.102813.

