# Supplementary material for: Tracing Nitrogen Flows Associated with Beef Supply Chains: A Consumption-Based Assessment
Source: Environ Sci Technol. 2024 Aug 2;58(32):14214–24. doi: 10.1021/acs.est.4c01651 (PMC11325653; doi:10.1021/acs.est.4c01651)
Supplement: Supplementary file 1 — es4c01651_si_001.pdf [file es4c01651_si_001.pdf]

SUPPORTING INFORMATION

Tracing nitrogen flows associated with beef supply chains: a consumption-based perspective

Anaís Ostroski[1], Oleg A Prokopyev[2], and Vikas Khanna[1,3*]

[1]Department of Civil and Environmental Engineering, University of Pittsburgh, 742 Benedum Hall, 3700 O'Hara Street, Pittsburgh, Pennsylvania 15261, United States

[2]Department of Industrial Engineering, University of Pittsburgh, 1025 Benedum Hall, 3700 O'Hara Street, Pittsburgh, Pennsylvania 15261, United States

[3]Department of Chemical and Petroleum Engineering, University of Pittsburgh, 3700 O'Hara Street, Pittsburgh, Pennsylvania 15261, United States

[*]Address correspondence to: khannav@pitt.edu

Table of Contents

Data 3

Cattle management and feed requirements 3

Fertilization 7

Results 8

Nitrogen intake and excretion 8

Nitrogen losses 8

Data

Cattle management and feed requirements

The feed requirements have been characterized by region through a series of surveys by Asem-Hiablie et al.[1–4] for 7 regions (Figure S1).

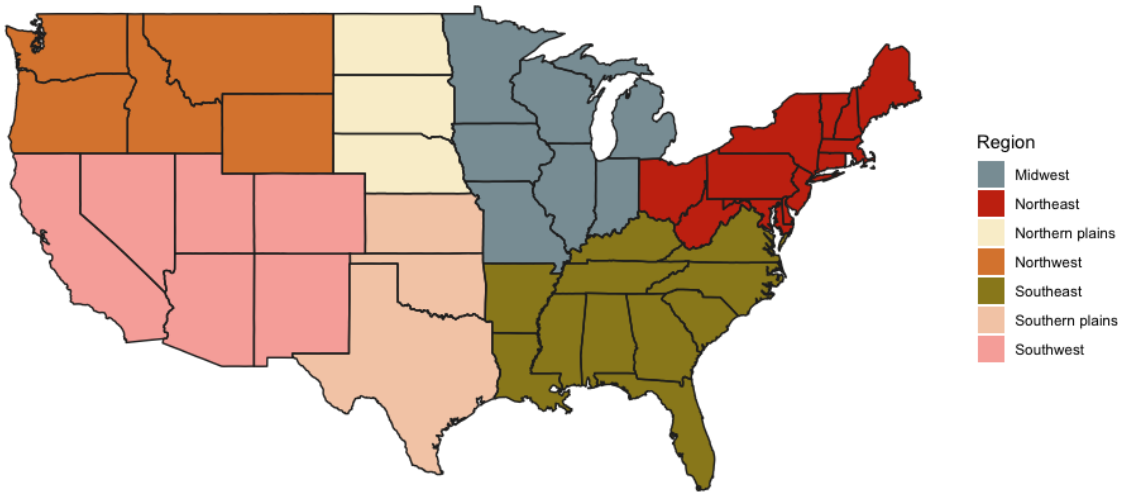

Figure S1: United States map of the 7 regions in this study

Cattle management parameters were compiled in Table S*1* and include initial body weight, ending body weight, amount of dry matter intake per animal per day, number of days in each stage, and percentage of cattle backgrounded and stocked [1–4]. Background and stocker refer to the phase between weaning and finishing, but in stocking the cattle is in open lot on grazing land, whereas when backgrounding, the cattle is at the feedlot, predominantly in confinement. We do not consider stocking due to lack of data. We assume calves are born weighting 35 kg[5] and their final weight depends on the destination and backgrounding rate, being either the initial body weight of backgrounds or finishing.

Table S1: Cattle management parameters per phase and region, where iBW is initial body weight, eBW exiting body weight, DM is daily matter. Source: Asem-Hiablie et al. [1–4]

Class	Region	iBW	eBW	Feed*	Period	Backgrounded	Stocked
		kg	*kg*	*kg DM/day*	*Days*	*% cattle*	*% cattle*
Cow-calf	Northern Plains			28.5	226.5		
	Midwest			26.2	226.5		
	Northwest			28.8	231		
	Southwest			28.3	231		
	Northeast			30.1	207		
	Southeast			21.4	237		
	Southern Plains			23.1	226.5		
Stocker	Northern Plains						3%
	Midwest						2%
	Northwest						4%
	Southwest						1%
	Northeast						6%
	Southeast						43%
	Southern Plains						7%
Background	Northern Plains			8.9	98	50%	
	Midwest			7.7	88	20%	
	Northwest	277	392	9.8	98	71%	
	Southwest	298	383	9.8	82	69%	
	Northeast			8.6	134	65%	
	Southeast	223	336	8.0	108	76%	
	Southern Plains			9.1	60	15%	
Finishing	Northern Plains	303	612	10.9	137		
	Midwest	290	617	10.5	175		
	Northwest	392	632	11.4	161		
	Southwest	383	612	8.4	95		
	Northeast	304	608	10.9	164		
	Southeast	336	502	10.1	154		
	Southern Plains	326	581	10.0	149		

For ranches, additional information includes purchased forage, purchased concentrate and stocking rate (Table S2) The difference between dry matter intake, purchased forage, and purchased

concentrate was assumed to be intake as pasture. Pasture yield was obtained from Kannan et al.[6] for cool season grasses (13.7 t/ha), warm season grasses (11.2 t/ha). Purchased forage was assumed to be alfalfa hay and purchased concentrate a mix of soybeans and corn grain. However, soybeans were consumed in low amounts due to nutrient content and lack of competitive prices[7].

Table S2: Purchased forage and concentrate, and stocking rate for ranches. Source: Asem-Hiablie et al. [1–4]

Region	Purchased forage	Purchased concentrate	Cow-calf stocking rate	Stocker stocking rate
	kg DM/animal/day	*kg DM/animal/day*	*ha/cow-calf pair*	*ha/stocker*
Northeast	3.3	0.59	1	0.8
Southeast	3.53	0.59	1	0.6
Northern Plains	2.8	0.94	4.3	2.5
Midwest	3.9	1.39	1.14	0.77
Northwest	1.97	0.36	13	7.8
Southwest	1.72	0.44	17.4	9.6
Southern Plains		0.6		
Kansas			3.8	1.3
Oklahoma			3.2	1.3
Texas			8	4

Feed intake for feedlot animals, in both backgrounding and finishing phases is shown in Table S3.

Table S3: Dry Matter intake for cattle in background and finishing phases within feedlots. Source: Asem-Hiablie et al. [1–4]

		% DM intake					
Class	Region	Corn Silage	Corn Grain	Distillers Grain	Alfalfa Hay	Mineral	Other
Background	Northeast	44.0	19.0	6.0	25.0	1.1	5.0
	Southeast	10.0	14.0	14.0	37.0	2.1	23.0
	Northern Plains	17.1	19.1	29.5	22.7	2.7	8.8
	Midwest	22.2	26.6	15.7	21.6	2.0	11.8
	Northwest	48.0	19.0	15.0	10.0	1.8	5.0
	Southwest	12.0	21.0	5.4	43	2.0	18.0
	Southern Plains	15.8	27.8	38.1	18.3	2.7	2.0
Finishing	Northeast	29.0	45.0	6.0	6.0	1.5	13.0
	Southeast	13.0	18.0	17.0	38.0	2.4	12.0
	Northern Plains	5.4	49.7	26.4	8.2	2.4	7.8
	Midwest	11.4	51.1	19.9	3.9	2.2	11.4
	Northwest	12.0	43.0	10.0	14.0	1.0	20.0
	Southwest	4.0	64.0	9.0	9.0	2.9	10.0
	Southern Plains	6.2	68.8	9.1	4.7	3.7	7.1

For manure management, we adopt the definitions by IPCC[8] and the EPA[9].

Table S4: Manure management definitions, adapted from the IPCC 2019 refinement of the 2006 Guidelines for National Emissions Inventories, Volume 4, Chapter 10, table 10.18.

System	Definition
Pasture/Range/Paddock (PRP)	The manure produced by cattle in pasture, here in cow-calf systems located in ranches, is allowed to lie as deposited.
Drylot	Open confinement area without vegetative cover and no requirement for bedding for moisture control. Manure can be removed periodically to spread on land, or combined with other manure management systems.
Solid storage	Manure is stacked with bedding material and stored for a set period, usually a few months. Can also be covered or compacted.

Fertilization

Asem-Hiablie[1–4] also provides information on nitrogen application for pastureland as average application rate, percentage of land that receives nitrogen, and percentage of farms that apply nitrogen (Table S5).

Table S5: Nitrogen application and fertilization rates for pastureland. Source: Asem-Hiablie et al. [1–4]

Region	N application rate	Fertilizer used	Fertilizer use
	kg N/ha	% land	% of farms
Northern Plains	48.5	11.0	16.0
Midwest	57.3	43.1	60.7
Southern Plains	98.3	4.4	46.2
Northeast	79.3	29.4	43.1
Southeast	104.8	25.7	64.9

Northwest	87.0	0.3	14.2
Southwest	125.0	0.4	14.8

Results

Nitrogen intake and excretion

Nitrogen intake is in accordance with values reported in Reed et al.[10] and IPCC[11]. Backgrounding cattle had daily nitrogen intake of 144 ± 17.4 gN, a retention of 25 ± 5.6 gN and excretion of 118 ± 17.2 in comparison to heifer values of 156 ± 57.8, 13, and 143 ± 54 and steer values of 130 ± 53.7, 25, and 105 ± 44.9 from Reed et al.[10], respectively. We can also compare the beef cow results with lactating cows from Reed et al.[10]: we found an intake of 315 ± 63.3, retention 22 ± 4.4, and excretion 293 ± 58.9 to be compared with 423 ± 145 intake, 140 retention, and 292 ± 101, respectively. Though the nitrogen retention is much lower given the assumptions adopted from IPCC, the daily excretion values are in excellent agreement.

Nitrogen losses

The total reactive nitrogen losses of each phase at the point of animal operation and at the point of feed production are shown in Table S6. The numbers were calculated as the sum of reactive nitrogen losses due to volatilization, leaching, runoff and nitrous oxide emissions during the duration of each phase and summarized by grams of nitrogen per final kg of carcass weight produced.

Table S6: Reactive nitrogen losses associated with each phase at animal operation and at feed production sites. Values are expressed as g N/kg CW.

Region	Cow-calf		Background		Finishing	
	Animal operation	Feed production	Animal operation	Feed production	Animal operation	Feed production
Midwest	34.07	18.61	1.68	2.02	23.15	10.09

Northeast	28.94	17.87	12.68	13.71	24.44	24.48
Northern Plains	41.11	9.93	5.31	5.47	16.48	13.48
Northwest	31.50	4.12	8.39	8.82	21.93	10.47
Southeast	30.40	20.99	9.87	5.87	32.13	11.17
Southern Plains	33.41	8.28	1.23	1.17	23.57	11.88
Southwest	36.58	4.87	8.60	6.80	9.01	21.70
Average	33.72	12.10	6.82	6.27	21.53	14.75
Percentage	74%	26%	52%	48%	59%	41%

Comparison of consumption and production-based nitrogen loss

Figure S2 shows the comparison of county-level nitrogen losses from production and consumption. It is evident that the Southern and Northern Plains and part of the Midwest are mostly associated from actual nitrogen losses during production, while not consuming much beef and having lower consumption-based nitrogen losses. More populous areas, such as the coasts are associated with high consumption-based nitrogen losses.

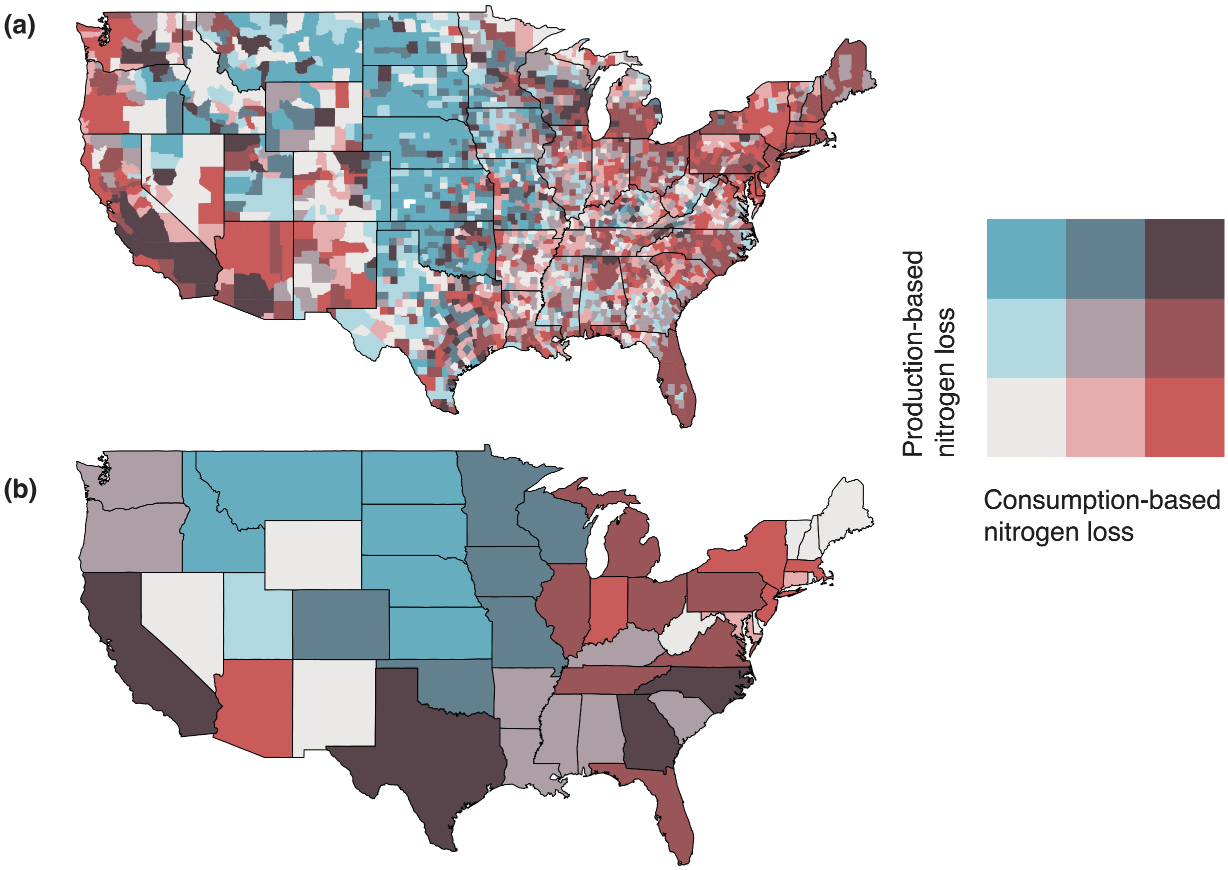

Figure S2: Bivariate distribution of nitrogen losses from beef production and consumption at (a) county-level and (b) state-level. Categories were assigned based on tertiles (33%, 66%, 100%), dividing the two variables in three groups (low, medium, high). Production tertiles cut-offs were 0.07, 0.6, and 25 Gg N for county-level and 11, 31, and 241 Gg N for state-level. Consumption tertiles were 0.06, 0.18, 34 Gg N for county-level and 11, 24, and 143 Gg N for state-level. The color scale indicates the combination of consumption and production respectively, explained by row: white (low-low), pink (medium-low), red (high-low), light blue (low-medium), gray (medium-medium), dark red (high-medium), teal (low-high), steel teal (medium-high), brown (high-high).

Nitrogen flows: sensitivity analysis

Nitrogen flows from the addition of manure to pastureland at 14 kg N/acre, and 55 kg N/acre.

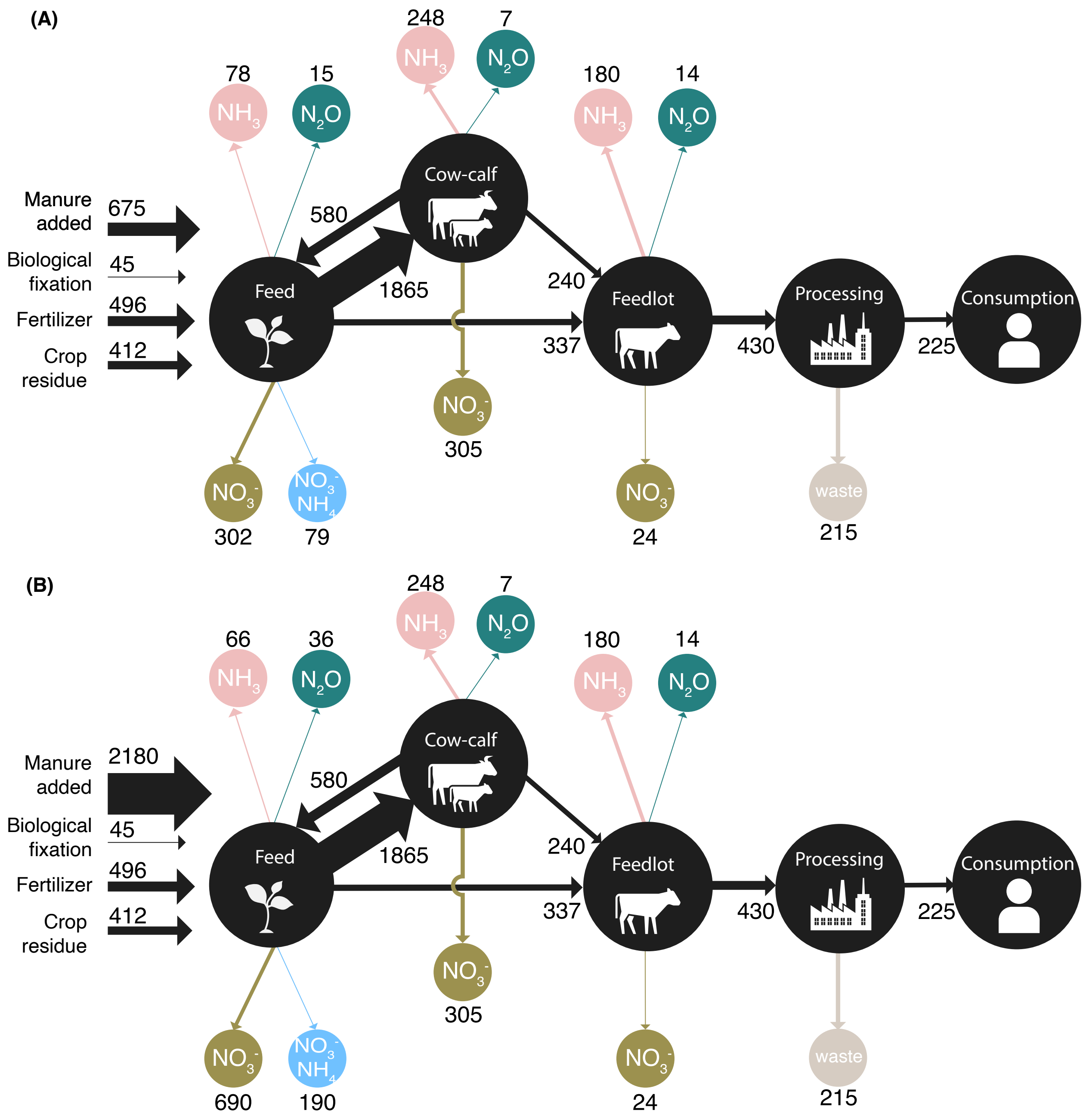

Figure S3: Nitrogen flows associated with beef supply chains. It shows the nitrogen retention and losses in animal feed, ranch (cow-calf), feedlot (backgrounding and finishing), processing, and consumption phases in Gg N for one year worth of beef consumption in the United States. All flows are for the year 2017. The scenarios include manure addition to pastureland with application rates of (a) 14, (b) 55 kg N/acre.

Nitrogen flows for the scenarios with the recycling of manure from feedlots at 14, 55 and 100 kg N/acre, respectively (Figure S4). These figures can be compared with Figure 3 in the main manuscript.

(A)

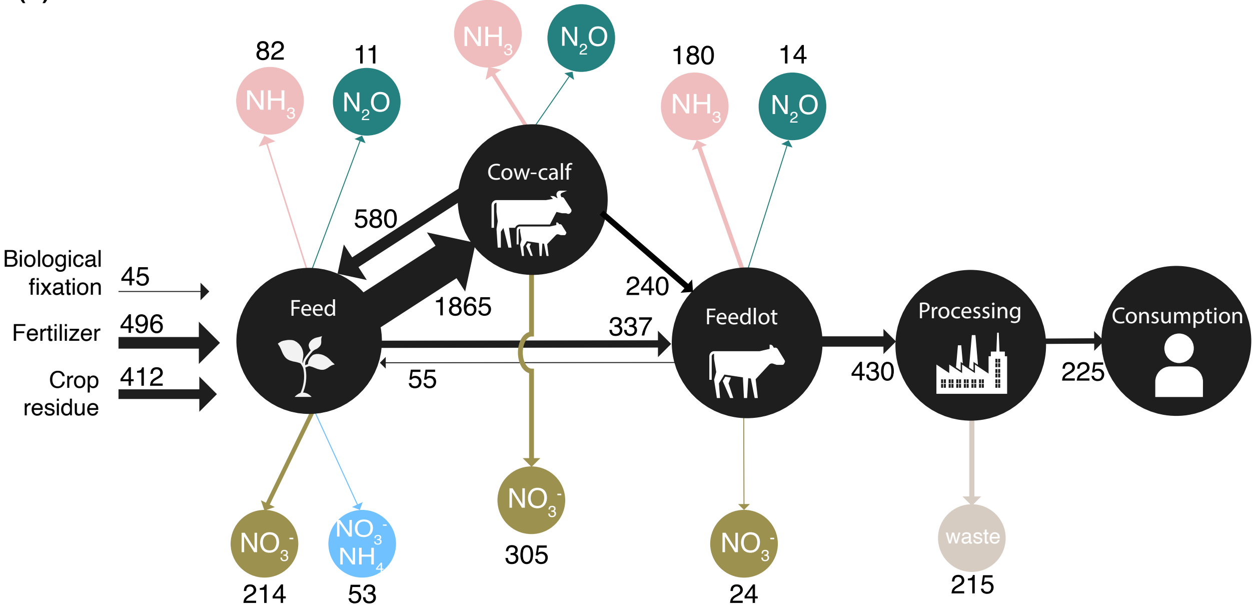

(B)

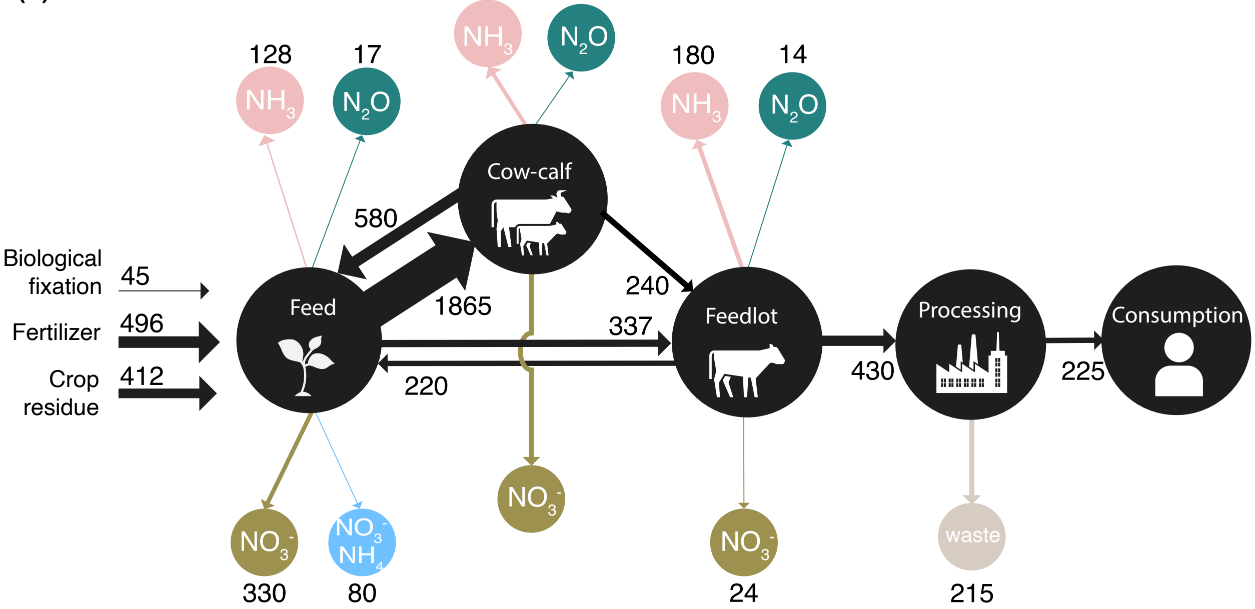

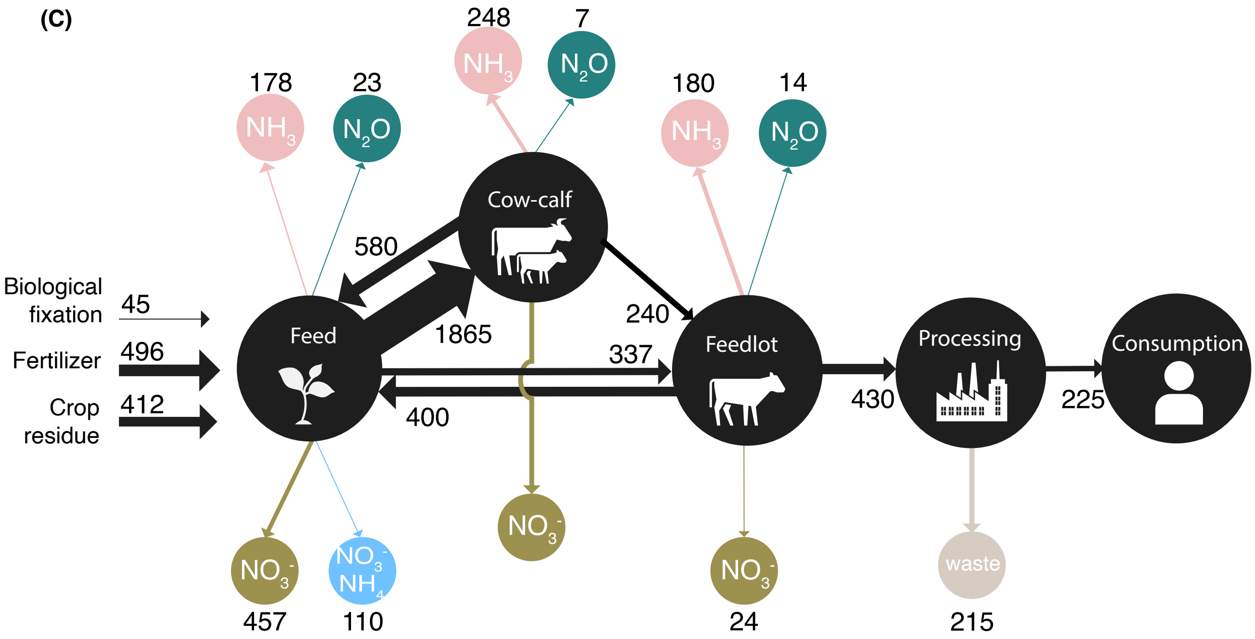

Figure S4: Nitrogen flows associated with beef supply chains. It shows the nitrogen retention and losses in animal feed, ranch (cow-calf), feedlot (backgrounding and finishing), processing, and consumption phases in Gg N for one year worth of beef consumption in the United States. All flows are for the year 2017. The scenarios include manure recycling from feedlots towards production of corn grain, corn silage, and alfalfa for feed with application rates of (a) 14, (b) 55, and (c) 100 kg N/acre.

References

1. Asem-Hiablie, S., Alan Rotz, C., Stout, R., Dillon, J. & Stackhouse-Lawson, K. Management characteristics of cow-calf, stocker, and finishing operations in Kansas, Oklahoma, and Texas. *Professional Animal Scientist* **31**, 1–10 (2015).
2. Asem-Hiablie, S., Rotz, C. A., Stout, R. & Stackhouse-Lawson, K. Management characteristics of beef cattle production in the Northern Plains and Midwest regions of the United States. *Professional Animal Scientist* **32**, 736–749 (2016).
3. Asem-Hiablie, S., Rotz, C. A., Stout, R. & Fisher, K. Management characteristics of beef cattle production in the western United States. *Professional Animal Scientist* **33**, 461–471 (2017).
4. Asem-Hiablie, S., Rotz, C. A., Stout, R. & Place, S. Management characteristics of beef cattle production in the eastern United States. *Professional Animal Scientist* **34**, 311–325 (2018).

5. Mekonnen, M. M., Neale, C. M. U., Ray, C., Erickson, G. E. & Hoekstra, A. Y. Water productivity in meat and milk production in the US from 1960 to 2016. *Environ Int* **132**, (2019).
6. Kannan, N., Osei, E., Gallego, O. & Saleh, A. Estimation of green water footprint of animal feed for beef cattle production in Southern Great Plains. *Water Resour Ind* **17**, 11–18 (2017).
7. Decision Innovation Solutions. *2017 Soybean Meal Demand Analysis: United States*. (2017).
8. IPCC. Volume 4: Agriculture, Forestry and Other Land Use. Chapter 10: Emissions form Livestock and Manure Management. *2019 Refinement to the 2006 IPCC Guidelines for National Greenhouse Gas Inventories* **4**, (2019).
9. EPA, U. S. *National Emission Inventory---Ammonia Emissions from Animal Agricultural Operations*. ftp://ftp.epa.gov/EmisInventory/2002finalnei/documentation/nonpoint/2002nei%5C_final%5C_nonpoint%5C_documentation0206version.pdf (2006).
10. Reed, K. F., Moraes, L. E., Casper, D. P. & Kebreab, E. Predicting nitrogen excretion from cattle. *J Dairy Sci* **98**, 3025–3035 (2015).
11. Lovelock, C. E. *et al. 2019 Refinement to the 2006 IPCC Guidelines for National Greenhouse Gas Inventories. Volume 4: Agricultura, Forestry and Other Land Use (AFOLU). Chapter 7: Wetlands*. vol. 4 (IPCC, Switzerland, 2019).
